# A simple, highly sensitive, and facile method to quantify ceramide at the plasma membrane

**DOI:** 10.1016/j.jlr.2022.100322

**Published:** 2022-12-20

**Authors:** Meaghan Greene, Maria Jose Hernandez-Corbacho, Anne G. Ostermeyer-Fay, Yusuf A. Hannun, Daniel Canals

**Affiliations:** 1 Department of Medicine, Stony Brook University, Stony Brook, NY, USA; 2 Stony Brook Cancer Center, Stony Brook University, Stony Brook, NY, USA; 3 Department of Biochemistry, Stony Brook University, Stony Brook, NY, USA

**Keywords:** lipid composition, cellular compartmentalization, plasma membrane ceramide, sphingosine, bacterial ceramidase, sphingolipid metabolism, subcellular organelles, doxorubicin, ceramide mass, ceramide hydrolysis, bSMase, bacterial sphingomyelinase, DGK, DAG kinase, PM-Cer, plasma membrane ceramide, pCDase, recombinant bacterial ceramidase, S1P, sphingosine 1-phosphate

## Abstract

The role of ceramide in biological functions is typically based on the elevation of cellular ceramide, measured by LC-MS in the total cell lysate. However, it has become increasingly appreciated that ceramide in different subcellular organelles regulates specific functions. In the plasma membrane, changes in ceramide levels might represent a small percentage of the total cellular ceramide, evading MS detection but playing a critical role in cell signaling. Importantly, there are currently no efficient techniques to quantify ceramide in the plasma membrane. Here, we developed a method to measure the mass of ceramide in the plasma membrane using a short protocol that is based on the hydrolysis of plasma membrane ceramide into sphingosine by the action of exogenously applied bacterial recombinant neutral ceramidase. Plasma membrane ceramide content can then be determined by measuring the newly generated sphingosine at a stoichiometry of 1:1. A key step of this protocol is the chemical fixation of cells to block cellular sphingolipid metabolism, especially of sphingosine to sphingosine 1-phosphate. We confirmed that chemical fixation does not disrupt the lipid composition at the plasma membrane, which remains intact during the time of the assay. We illustrate the power of the approach by applying this protocol to interrogate the effects of the chemotherapeutic compound doxorubicin. Here we distinguished two pools of ceramide, depending on the doxorubicin concentration, consolidating different reports. In summary, we have developed the first approach to quantify ceramide in the plasma membrane, allowing the study of new avenues in sphingolipid compartmentalization and function.

Ceramide regulates cell senescence, cell differentiation, apoptosis, cell cycle arrest (1), cell adhesion, and cell migration (2). The role of ceramide as a bioactive lipid has been established by demonstrating that changes in cellular ceramide levels are either sufficient or necessary to mediate these biological processes. However, modulating cellular ceramide to control these biological processes has been challenging for the last 30 years. Cellular ceramide levels result from the sum of ceramide generated by distinct enzymatic pathways (de novo, salvage, lysosomal, and hydrolytic. Reviewed in (1)). Different research groups have reported targeting individual pathways in order to block a specific biological response. However, there is a lack of clarity about which ceramide-generating pathways regulate which biological outcomes. During the last decade, a proposed concept, the 'many ceramides' hypothesis (3), has been gaining support. This hypothesis postulates that different species of ceramide, based on modifications of ceramide structure and their location in distinct subcellular compartments, might mediate different processes.

In agreement with this hypothesis, elevation in ceramide has been shown to occur in specific membranes, such as the mitochondria (4, 5, 6, 7, 8), in the Golgi apparatus (9, 10, 11), and in the plasma membrane (2, 12). A few studies have attempted to detect these local ceramides by using differential centrifugation for lysosomes (13) and mitochondria (14, 15). Purer membranes have been obtained by further purification by gradient centrifugation, and the ceramide has been measured by either LC-MS/MS (16, 17) or radiolabeling (18). These studies demonstrated that ceramide signaling is compartmentalized. Moreover, ceramide antibodies have emerged as powerful tools to detect cellular ceramide. They have been used to report on changes in ceramide levels in the plasma membrane (2, 12), the nucleus (19), and intracellular membranes (20), providing more evidence that ceramide signaling is compartmentalized (16).

More profound studies on specific subcellular ceramides would require tools to detect ceramide and quantitative measurements. Here, we describe a method to measure plasma membrane ceramide (PM-Cer) with minimal sample manipulation by hydrolyzing PM-Cer with recombinant bacterial ceramidase (pCDase) and measuring the sphingosine produced. After a short exposure to pCDase, PM-Cer is calculated as the 1:1 M ratio of hydrolyzed ceramide to produce sphingosine. Metabolic effects of ceramide depletion and sphingosine formation are blocked by the chemical fixation of cells without altering the sphingolipid composition and membrane integrity.

As an example of the use of this method, we have detected two distinguishable pools of ceramide in response to doxorubicin treatment: One at the plasma membrane and one intracellular when low and high doses of doxorubicin were used, respectively.

The method presented in this work represents a significant qualitative step in plasma membrane detection and quantification. In contrast to previous methods (cell fractionation and ceramide antibody), this method allows the determination of PM-Cer in a large number of samples (potentially could be used in high throughput screening), lowering the detection limit to a few pmols of ceramide per 1 Million cells, and following ceramide generation in a time-course and dose-dependent manner. In contrast to other reported methods to detect PM-Cer, this method is also simple to apply, does not need skilled personnel, and is highly reproducible.

In summary, we have developed a protocol that allows the measurement of PM-Cer with high sensitivity, compatible with acute signaling and which otherwise would not be detectable using conventional protocols.

## MATERIAL AND METHODS

### Reagents

Doxorubicin was from Sigma-Aldrich (St. Louis, MO). Methanol and water solvents for LC-MS analysis were purchased from Fisher Scientific (Waltham, MA). HLPC column Spectra 3 μm C8SR column (3 μm particle, 150 × 3.0 mm, #S-3C8SR-FJ) was from Peeke Scientific (Redwood City, CA). Internal standards and standards of sphingolipids used in LC-MS were obtained from the Biological Mass Spectrometry Core, Stony Brook University (Stony Brook, NY) and from Avanti Polar Lipids (Alabaster, AL).

### Cell culture

HeLa cells (ATCC, Manassas, VA) were fed with Dulbecco's Modified Eagle Medium high glucose from Thermo Fisher (Waltham, MA) supplemented with 10% of fetal bovine serum (Thermo Fisher). MCF-7 cells (ATCC) were fed with RPMI 1640 medium, supplemented with 10% of fetal bovine serum. Authentication of the cell lines was performed by ATCC Cell Line Authentication Service, matching 100% with HeLa and 93% for MCF-7 in the ATCC reference Database Profile. The presence of mycoplasma was tested on a monthly basis. Cells were cultivated in a humidified incubator at 37°C in a 5% CO2 atmosphere.

### Recombinant protein expression and purification

Bacterial sphingomyelinase from *Bacillus cereus*, bacterial ceramidase from *Pseudomonas aeruginosa,* and lysenin from *Eisenia fetida* were expressed as his-tag fusion protein in *E. coli* BL21, and protein was purified using affinity chromatography with a HisTrap FF 5 ml column and size-exclusion column (Superdex 200 HiScale 26/40) using a FPLC system Äkta Pure 25 from Cytiva (Marlborough, MA), as previously described (21).

### Sphingolipid measurement

Sphingolipids from cells (500K) were extracted using ethyl acetate: 70% isopropanol 3:2 mix (22). Internal standards were added to each sample (50 pmols) before extraction. Extracted lipids were resuspended in 150 ul methanol, and 5 ul were injected in an Agilent Infinity 1260–6120 LC-MS system (Santa Clara, CA). Injection volume: 5ul; Flow: 0.5 ml/min; Mobile phase A: Fisher Water Optima LC/MS, 1 mM ammonium formate, 0.2% formic acid; Mobile phase B: Fisher Methanol Optima, 1 mM ammonium formate, 0.2% formic acid. Buffer pre-heated 50°C; Gradient: 0–1 min 80%B, 1–7.0 min 99%B, 7–16 min 99%B. Parameters were set as peak width 0.02 min, step size 0.2, fragmentor 220 V, positive polarity, drying gas 10 L/min, nebulizer 30 psi, drying gas temperature 350C, and capillary voltage (Vcap) +5000V. Transition masses are sphingosine m/z (300.3), sphingosine 1 phosphate (380.4), ceramide d18:1/16:0 (520.4), ceramide d18:1/24:1 (630.7), hexosylceramide d18:1/16:0 (700.4), hexosylceramide d18:1/24:1 (810.4), sphingomyelin d18:1/16:0 (703.4), and sphingomyelin d18:1/24:1 (729.4). Internal standards are sphingosine d17:1 (286.2), sphingosine 1-phosphate (S1P) d17:1 (366.1), ceramide d17:1/16:0 (506.4), ceramide d17:1/24:1 (616.6), hexosylceramide d18:one-eighth:0 (588.4), and sphingomyelin d18:1/17:0 (717.6).

### Confocal microscopy

HeLa cells were grown in gamma-irradiated, 35 mm glass-bottom poly-D-lysine coated dishes (P35GC-1.5-10-C; MatTek Corp. Ashland, MA) at 10^5^ cells/ dish. Cells were fixed with 4% paraformaldehyde in PBS for 20 min and visualized using a Leica TCS SP8 confocal microscope with HC PL APO 63x/1.40 oil immersion objective (Morrisville, NC). Raw data files (.lif) were generated using Leica Application Suite X, and images were extracted using Bio-formats [OME] (openmicroscopy.org) package for python (python-bioformats).

### Software

Mass Hunter Quantitative Analysis for LCMS version 10.1 (Agilent Technologies, Santa Clara, CA) was used to quantify sphingolipids from LC-MS raw files. Bibliographic references were managed using Mendeley desktop v 1.19.4 software (mendeley.com) and EndNote 20 (endnote.com). Editing and data processing for this manuscript used free open source software (FOSS) whenever possible, including Debian Linux (debian.org), LibreOffice (libreoffice.org), GIMP (gimp.org), Python (python.org), Spyder (spyder-ide.org), and OpenCV (opencv.org). GraphPad Prism 9.3.1 was used for plotting the final results for the manuscript (GraphPad Software, San Diego, CA, graphpad.com).

## RESULTS

### Slow clearance of ceramide from the plasma membrane

In previous work, we used bacterial sphingomyelinase (bSMase) from *Bacillus cereus* to generate ceramide at the plasma membrane. We showed that 100 mU of bSMase for 10 min is enough to hydrolyze the entire sphingomyelin content in the plasma membrane without modifying the intracellular pools of sphingomyelin (21). In addition, we found that PM-Cer did not move to ER, Golgi apparatus, or nuclear intracellular membranes (23) nor was it metabolized to other sphingolipid species within a window of 10 min upon its generation in HeLa cells. However, within this time frame, and hydrolyzing as little as 5%–10% of the plasma membrane sphingomyelin, it was sufficient to trigger the dephosphorylation of the ezrin-radixin-moesin family of proteins, strongly suggesting that ceramide located at the plasma membrane was bioactive (23). Thus, it became important to understand the kinetics and metabolism of PM-Cer. To that end, we required methods to detect and quantify PM-Cer.

**Fig. 1:**
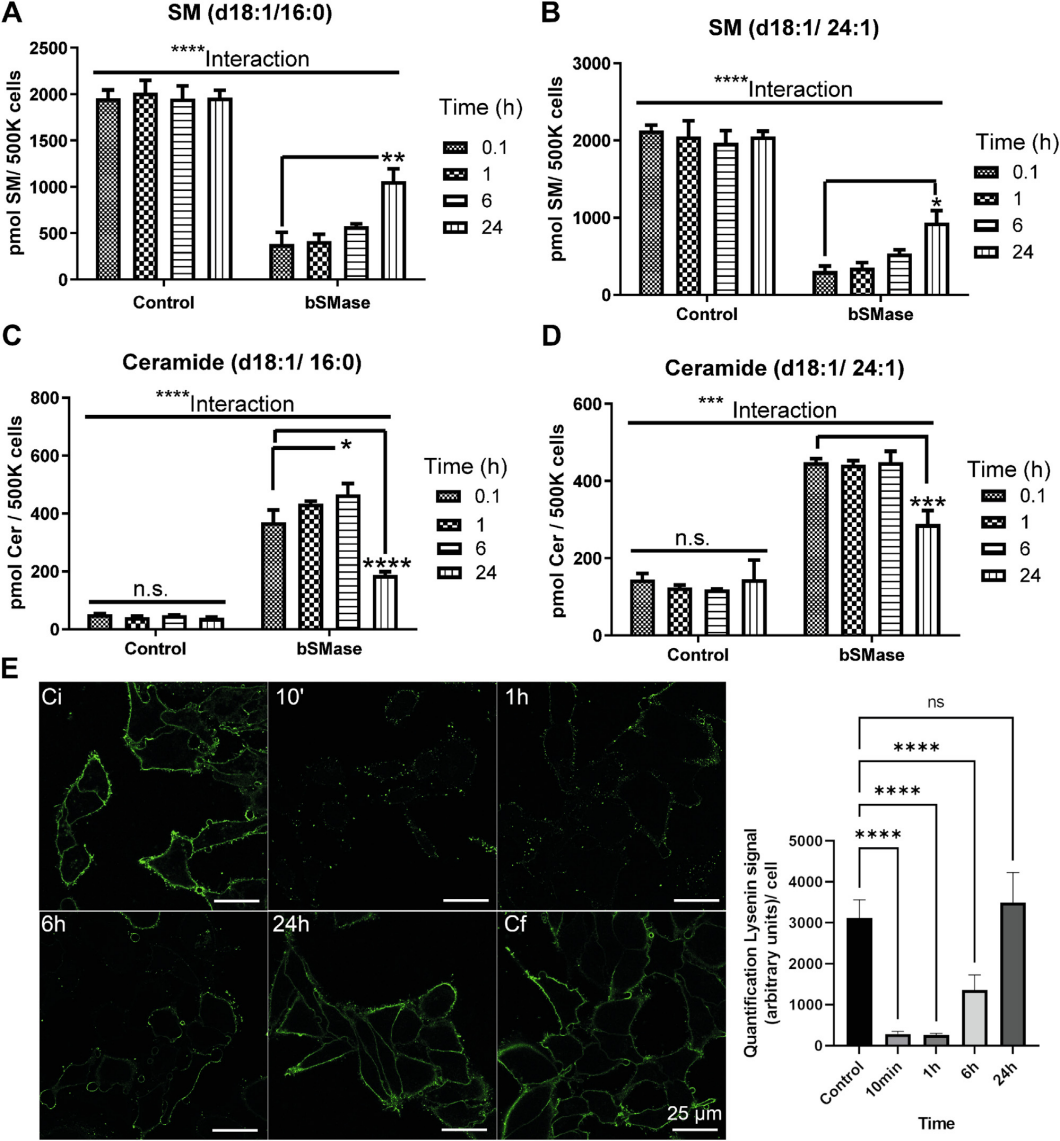
After plasma membrane sphingomyelin hydrolysis, recovery of sphingomyelin and ceramide levels starts between 1 and 6 h after bSMase treatment. HeLa cells were treated with bSMase (100 mU/ml) or vehicle (control) for 10 min. Cells were washed to remove bSMase from the media, and sphingolipid content was measured by LC-MS at different times. For simplicity, only the two major sphingolipid species, d18:1/16:0, and d18:1/24:1 were measured. (A) SM (d18:1/16:0), (B) SM (d18:1/24:1), (C) ceramide (d18:1/16:0), and (D) ceramide (d18:1/24:1). (E) HeLa cells were treated with vehicle (Ci- control at time zero, Cf- control at 24 h), or treated with bSMase for 10 min, cells were washed, and PM SM was visualized at different times using venus-lysenin binding protein. Quantification of independent experiments (n = 6) is also shown. SM, sphingomyelin. Statistics: Two-way ANOVA post hoc Tukey test ∗*P*<0.05; ∗∗*P*<0.01; ∗∗∗*P*<0.001; ∗∗∗∗*P*<0.0001. bSMase, bacterial sphingomyelinase; SM, sphingomyelin.

In order to evaluate the production of PM-Cer, HeLa cells were treated with bSMase for 10 min, then bSMase was removed from the cell culture media, and sphingomyelin (Fig. 1A, B) and ceramide (Fig. 1C, D) were measured at different time points (0, 1, 6, and 24 h) by mass spectrometry. As reported previously, a large percentage of cellular sphingomyelin was hydrolyzed at 10 min of bSMase treatment. Partial recovery of sphingomyelin and ceramide were detected only at the 24 h time point, suggesting that changes of sphingomyelin and ceramide in the plasma membrane of HeLa cells were stable for at least 6 h, and re-synthesis of sphingomyelin and further metabolism of the newly generated ceramide had to start between 6 and 24 h time. Of note, a trend of sphingomyelin recovery was detected already at 6 h, although it was not statistically significant.

To confirm the recovery of sphingomyelin at the plasma membrane and to use an independent approach, plasma membrane sphingomyelin content was evaluated using the fluorescent-labeled binding protein lysenin (21, 24) (Fig. 1E). Using this method, hydrolysis of the totality of plasma membrane sphingomyelin by bSMase occurred at 10 min upon bSMase, an initial recovery was detected at 6 h, and a robust recovery was visualized at 24 h, confirming the results using mass spectrometry.

The results from the two techniques suggested that changes in sphingomyelin and ceramide in the plasma membrane are stable, at least during the first hour.

### Sphingosine and complex sphingolipids are generated after 6–24 h of plasma membrane ceramide production

**Fig. 2:**
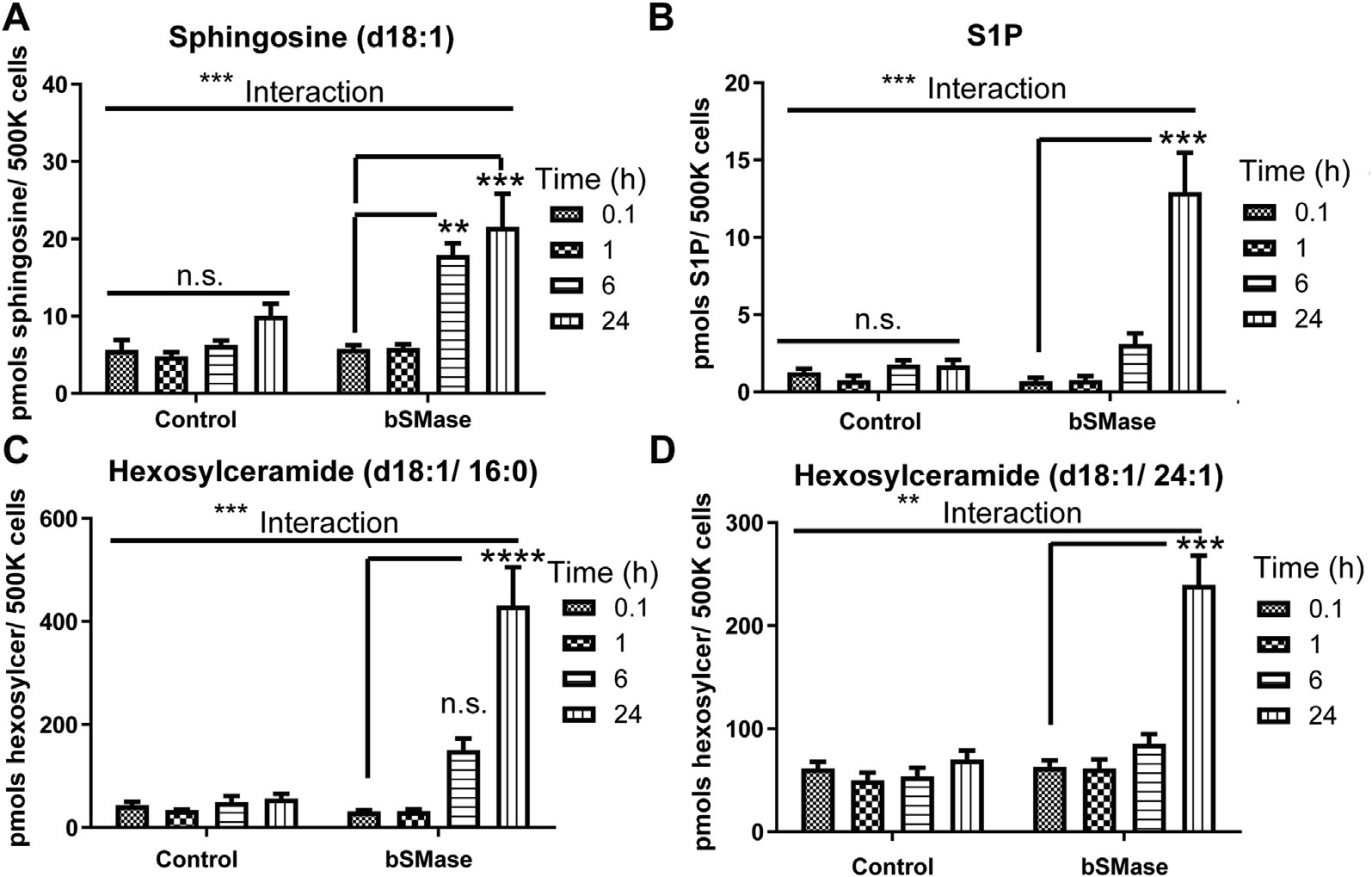
PM-Cer regeneration. HeLa cells were treated with bSMase (100 mU/ml) for 10 min, washed, and cells were collected at different times, and lipids were extracted and quantified by LC-MS. (A) sphingosine, (B) S1P, (C) hexosylceramide (d18:1/16:0), and (D) hexosylceramide (d18:1/24:1). Statistics: Two-way ANOVA post hoc Tukey test. n.s. = not significant; ∗∗*P*<0.01;∗∗∗ *P*<0.001. bSMase, bacterial sphingomyelinase; PM-Cer, plasma membrane ceramide; S1P, sphingosine 1-phosphate.

Besides forming sphingomyelin, ceramide can follow other fates. Interestingly, a previous study on mammalian neutral ceramidase nCDase (25) suggested that in cells that overexpress recombinant nCDase, this enzyme can couple to ceramide produced by bSMase to metabolize it to sphingosine and sphingosine-1phosphate. In addition, ceramide and sphingosine could be recycled to complex sphingolipids in the Golgi apparatus, such as glycosphingolipids. In the previous experiment, we observed PM-Cer being cleared after 6–24 h upon its generation. To further analyze the kinetics of PM-Cer conversion to other lipids, we monitored sphingosine, S1P, and hexosylceramide, the last as a readout of the first step to the glycolipid synthesis. Sphingosine generation was not detected at 1 h but was detected at 6 h and further increased at 24 h (Fig. 2A), while sphingosine 1-phosphate and hexosylceramides were robustly detected at 24 h; there were apparent increases at 6 h, but these did not reach statistical significance. These results confirmed that within 1 h of its generation at the plasma membrane, ceramide is not being metabolized to any of its most common metabolites. From these results, we decided to define 1 h windows as the maximum time after ceramide elevation to quantify its levels.

### Sphingosine generated from artificially hydrolyzed plasma membrane ceramide is rapidly metabolized

**Fig. 3:**
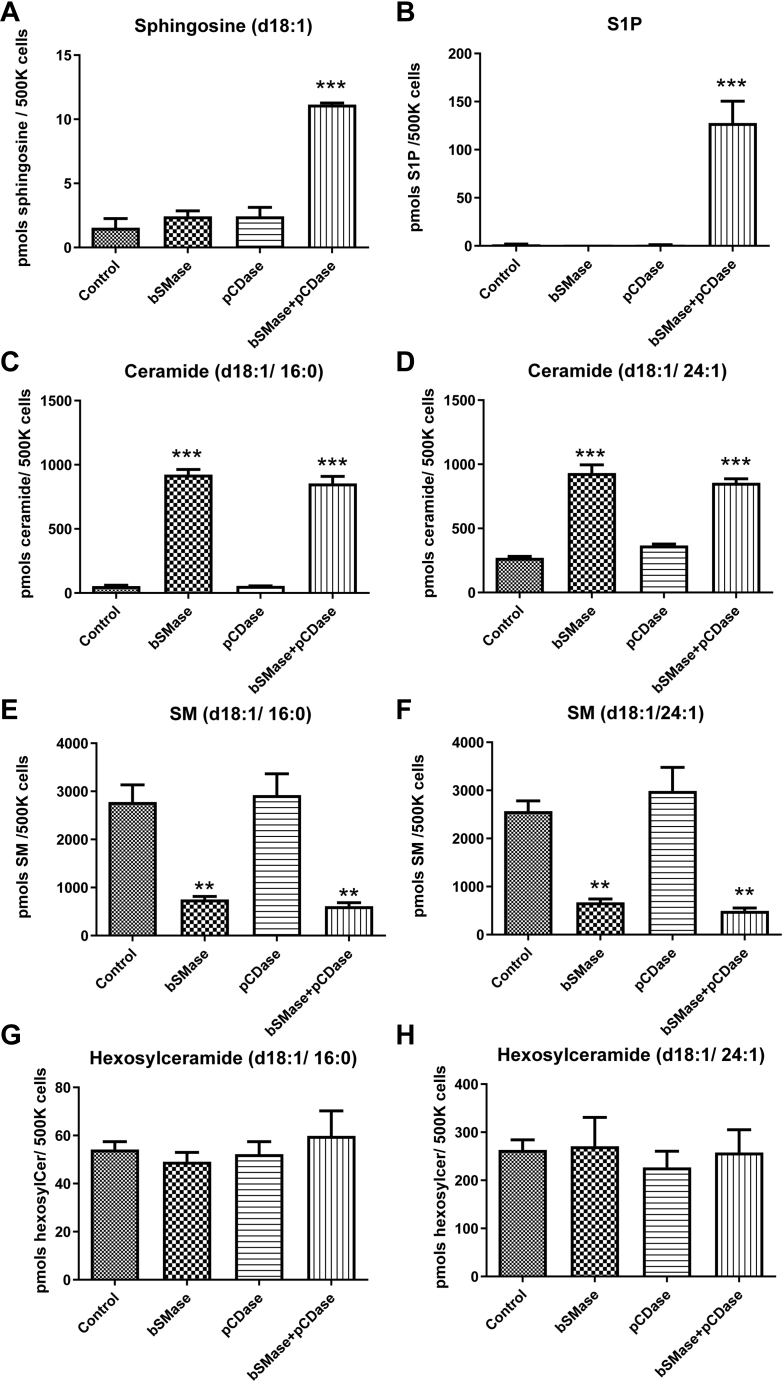
Exogenously applied bacterial pCDase hydrolyzes PM-Cer to sphingosine, which is quickly metabolized to S1P. HeLa cells were treated with bSMase (100 mU/ml), pCDase (10 ug/ml), or a combination of both for 10 min. Cellular lipids were extracted, and (A) sphingosine, (B) S1P, (C) ceramide (d18:1/16:0), (D) ceramide (d18:1/24:1), (E) SM (d18:1/16:0), (F) SM (d18:1/24:1), (G) hexosylceramide (d18:1/16:0), and (H) hexosylceramide (d18:1/24:1) were quantified by LC-MS. SM, sphingomyelin. One-way ANOVA post hoc Tukey test ∗∗ *P*<0.01; ∗∗∗ *P*<0.001. bSMase, bacterial sphingomyelinase; PM-Cer, plasma membrane ceramide; pCDase, recombinant bacterial ceramidase; S1P, sphingosine 1-phosphate.

The elevation in PM-Cer might not be statistically significant when measured in a total cell lysate because of the high intracellular ceramide amount. However, if this PM-Cer could be converted into sphingosine and due to the lower levels of sphingosine in cells, the elevation in sphingosine could be used to estimate the amount of PM-Cer. pCDase from *Pseudomonas aeroginosa* hydrolyzes ceramide into sphingosine and free-fatty acid (26, 27), and we have previously shown that when pCDase is applied to the cell culture medium, it can hydrolyze ceramide in the plasma membrane (21). In order to estimate how much hydrolyzed ceramide is converted in sphingosine, the latter should be, ideally, stable and not metabolized, at least during the duration of the assay. However, sphingosine can be phosphorylated by sphingosine kinases to S1P (21, 28). Even worse, S1P provides for the exit of the sphingoid backbone from the sphingolipid network, and this occurs by the action of the S1P lyase to form hexadecenal and phosphoethanolamine (29), meaning sphingosine could be dramatically reduced by exiting the sphingolipids pathway. Sphingosine can also be re-acylated to ceramide by six different ceramide synthases and be further metabolized to complex sphingolipids (30). The dynamics and metabolism of newly generated sphingosine at the plasma membrane have not been characterized. To address this point, HeLa cells were treated with bSMase for 10 min to generate PM-Cer, the cell media were replaced with fresh serum-free media, and 100mU of pCDase was applied to the culture media for 10 min. Sphingosine production was measured to validate pCDase activity in the cell culture (Fig. 3A). However, pCDase was able to generate S1P (Fig. 3B). The sphingolipid profile of commonly studied sphingolipids was also analyzed. As expected, bSMase produced a large amount of ceramide (Fig. 3C). However, the results from using pCDase were unexpected: pCDase only reduced a small amount of newly generated ceramide, and it was not even statistically significant. We did not detect changes in the sphingomyelin and hexosylceramide content upon sphingosine production (Fig. 3E–H), suggesting that sphingosine generated from the hydrolysis of PM-Cer is not recycled to these sphingolipids within the first hour. This rapid metabolism made impossible the use of sphingosine as a read-out for PM-Cer.

### Blocking sphingolipid metabolism by chemical fixation does not alter plasma membrane sphingolipid profile

**Fig. 4:**
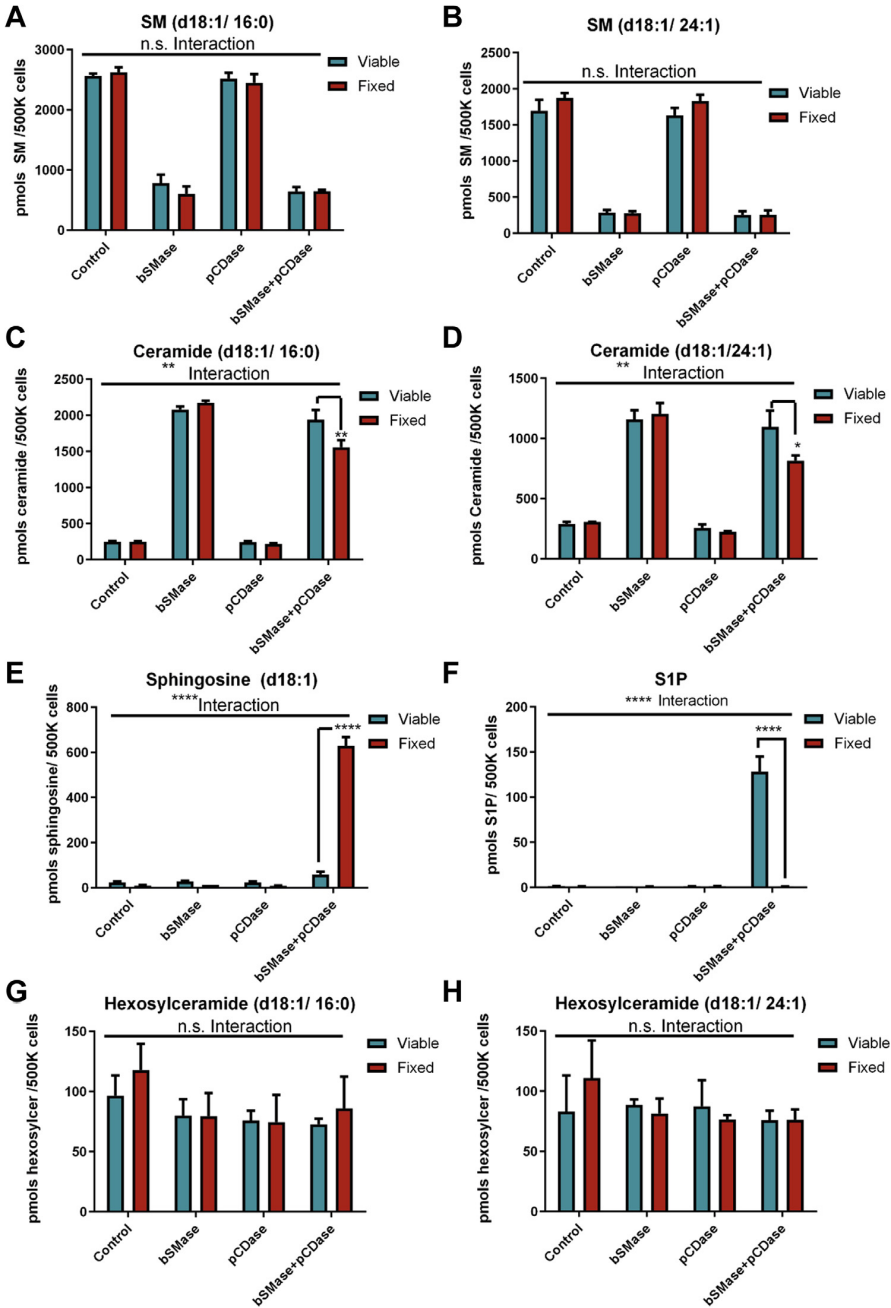
Chemical fixation of cells does not change sphingolipid composition at the PM but blocks sphingosine metabolism. HeLa cells were washed and kept in serum-free media (Viable cells*, blue color*) or fixed with paraformaldehyde for 10 min (fixed, *red color*). Cells were washed with serum-free media and incubated with bSMase, pCDase, or both enzymes for 10 min. Cellular lipids were extracted and quantified by LC-MS, and the lipid amounts from viable cells and fixed cells were compared. (A) SM (d18:1/16:0), (B) SM (d18:1/24:1), (C) ceramide (d18:1/16:0), (D) ceramide (d18:1/24:1), (E) sphingosine, (F) S1P, (G) hexosylceramide (d18:1/16:0), and (H) hexosylceramide (d18:1/24:1). SM, sphingomyelin. Statistics: Two-way ANOVA post hoc Tukey test n.s. = not significant; ∗*P*<0.05; ∗∗ *P*<0.01; ∗∗∗∗*P*<0.0001. bSMase, bacterial sphingomyelinase; S1P, sphingosine 1-phosphate; pCDase, recombinant bacterial ceramidase.

Ideally, if sphingolipid metabolism and membrane trafficking could be blocked without altering the plasma membrane integrity, we could apply pCDase to estimate the PM-Cer content. Chemical fixation is extensively used to fix cells for imaging studies without disrupting the subcellular structure. However, chemical fixation, such as with paraformaldehyde, only crosslinks proteins and does not fix lipids. However, lipid staining protocols have been used in fixed cells (20, 31), and fixed cells need to be permeabilized with detergents to access the intracellular compartments (32). These points suggested that chemical fixation might not disrupt cellular membranes, at least for a short period of time. Thus, aiming to block all enzymatic activity and to stop any further metabolic flux, we fixed the cells with formaldehyde after 10 min of bSMase treatment. To test if the sphingolipid content was affected by the fixation procedure, we compared the sphingolipid profile before and after fixation. As depicted in Fig. 4, no differences were detected in sphingomyelin and ceramide with the use of bSMase in control versus fixed samples. To evaluate if the content in the plasma membrane could be altered, we treated cells with bSMase and compared the drop in sphingomyelin and the increase in ceramide production in intact versus fixed cells (Fig. 4A–D). Again, no differences were detected, suggesting that protein fixation does not alter the sphingolipid content in the plasma membrane.

Then, we repeated the pCDase experiment and analyzed the sphingosine content as well as its metabolic fate. As shown in Fig. 4E, F, fixation blocked S1P production. Ceramide hydrolysis with bSMase showed the same extent in unfixed and fixed cells, but the reduction with bCDase became evident after fixation (Fig. 4C, D). Importantly, sphingosine levels became considerably higher (Fig. 4E, F). We also confirmed that neither fixation nor any of the enzymatic treatments had any effect on hexosylceramide cellular levels (Fig. 4G, H).

These results suggested that fixing cells with 4% of paraformaldehyde did not interfere with the hydrolysis of PM-Cer into sphingosine; however, fixation blocked sphingosine metabolism into S1P. Therefore the formation of sphingosine could be used to measure PM-Cer content as the molar ratio of hydrolyzed ceramide: sphingosine production is 1:1.

### Determining optimum conditions for pCDase treatment in fixed cells

**Fig. 5:**
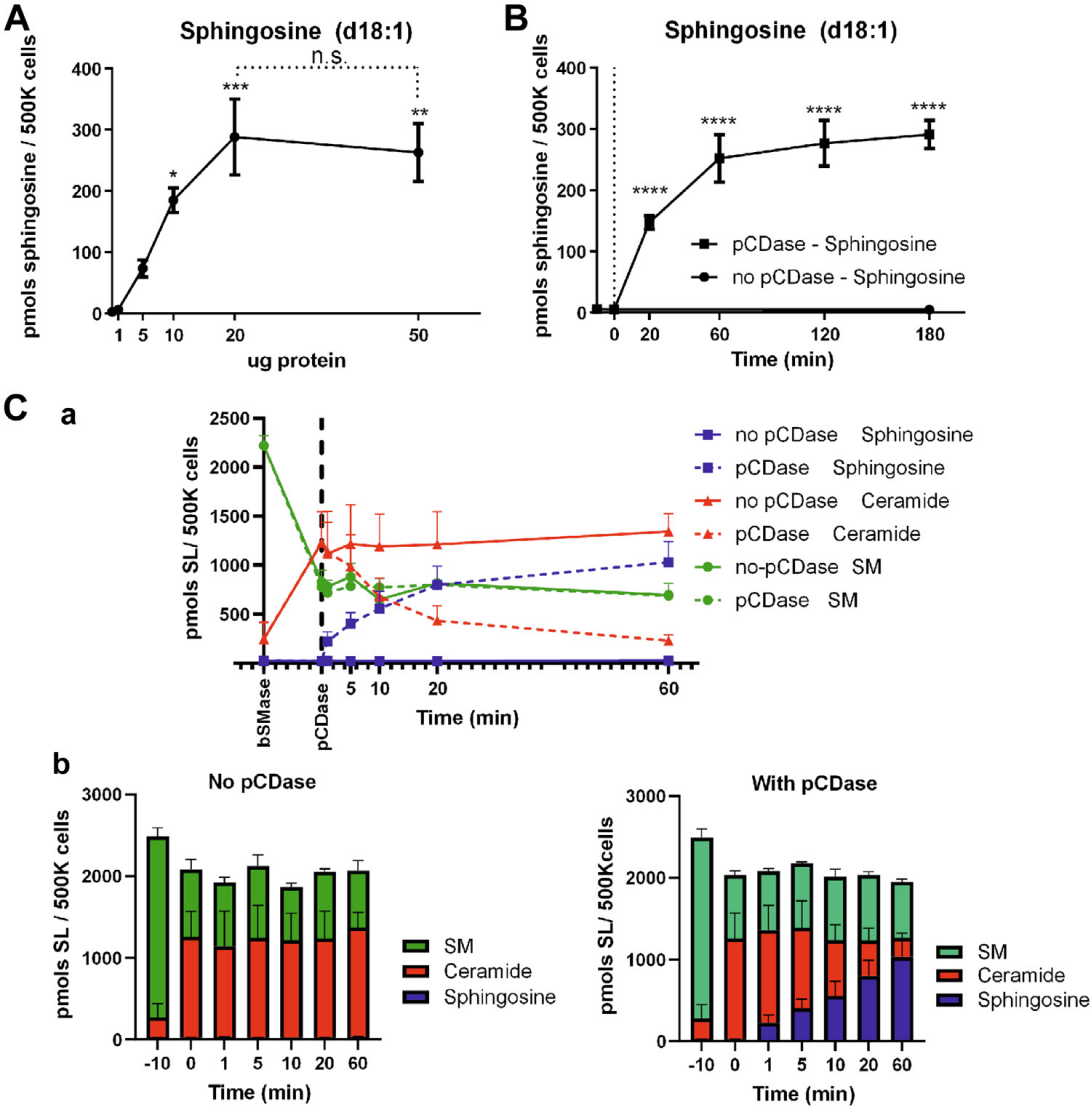
Dose and time optimization of pCDase treatment. HeLa cells were treated with bSMase (100 mU/ml) for 10 min, washed, fixed, and treated with (A) different amounts of pCDase and (B) different incubation times. Sphingosine production was monitored using LC-MS. C-a: HeLa cells were treated with bSMase, washed, and cells were fixed and treated with vehicle (*solid lines*) or pCDase (20ug pCDase/ 5 ml culture/ 60 mm diameter dish, *dashed lines*). Sphingolipids were extracted and analyzed by LC-MS. The amounts of the different sphingolipids were measured and plotted in the same graph. SM (sphingomyelin, *green*), ceramides (*red*), and sphingosine (*blue*). C-b: The total mol amount of sphingolipids remained constant during the assay, both with (right panel) or without treatment with pCDase (left panel). bSMase, bacterial sphingomyelinase; pCDase, recombinant bacterial ceramidase.

Initially, we used 10ug pCDase protein/ml for 5∗10ˆ5 cells/ml, as we have reported previously (21). We tested increasing amounts of pCDase (Fig. 5A), and the results showed that 20ug/ml of protein was saturating the system, and higher quantities did not improve the transformation kinetics. Therefore, we chose 20ug/ml of pCDase for the following studies.

The next step was to define the optimal time of treatment with pCDase in the assay. Previously, results were performed at 10 min. As shown in Fig. 5B, using 20ug pCDase at different times showed that at 60 min of treatment with pCDase, the slope of transformation reached a plateau.

It also became important to define the effects of the various treatments on the balance of the main metabolites (SM, ceramide, and Sph). Figure 5C shows the effects of the sequence of treatments. BSMase was applied to living cells, and after 10 min of incubation, cells were fixed and treated with pCDase for different times, up to 60 min. Levels of sphingomyelin (Fig. 5C(a), green), ceramide (red), and sphingosine (blue) were measured at different reported times with (dashed lines) or without pCDase (solid lines). The amount of sphingomyelin dropped after 10 min of bSMase treatment. It should be noted that there were no statistical differences in sphingomyelin levels during the time of the assay between samples treated with and without pCDase. In the absence of pCDase, there were no changes in the levels of SM, ceramide, or sphingosine since their levels remained constant after the initial change with bSMase (Fig. 5C(a) solid lines). When pCDase was applied, sphingosine increased with time, and this corresponded to a decrease of ceramide. In fact, the sum of sphingosine and ceramide remained nearly constant, demonstrating a 1:1 M ratio of the pmols sphingosine formed with the pmols of ceramide that was hydrolyzed (Fig. 5C(b)). These results demonstrate the effectiveness on pCDase in the near total conversion of PM-Cer to sphingosine under these optimized conditions.

### Sensitivity of detection of plasma membrane ceramide

**Fig. 6:**
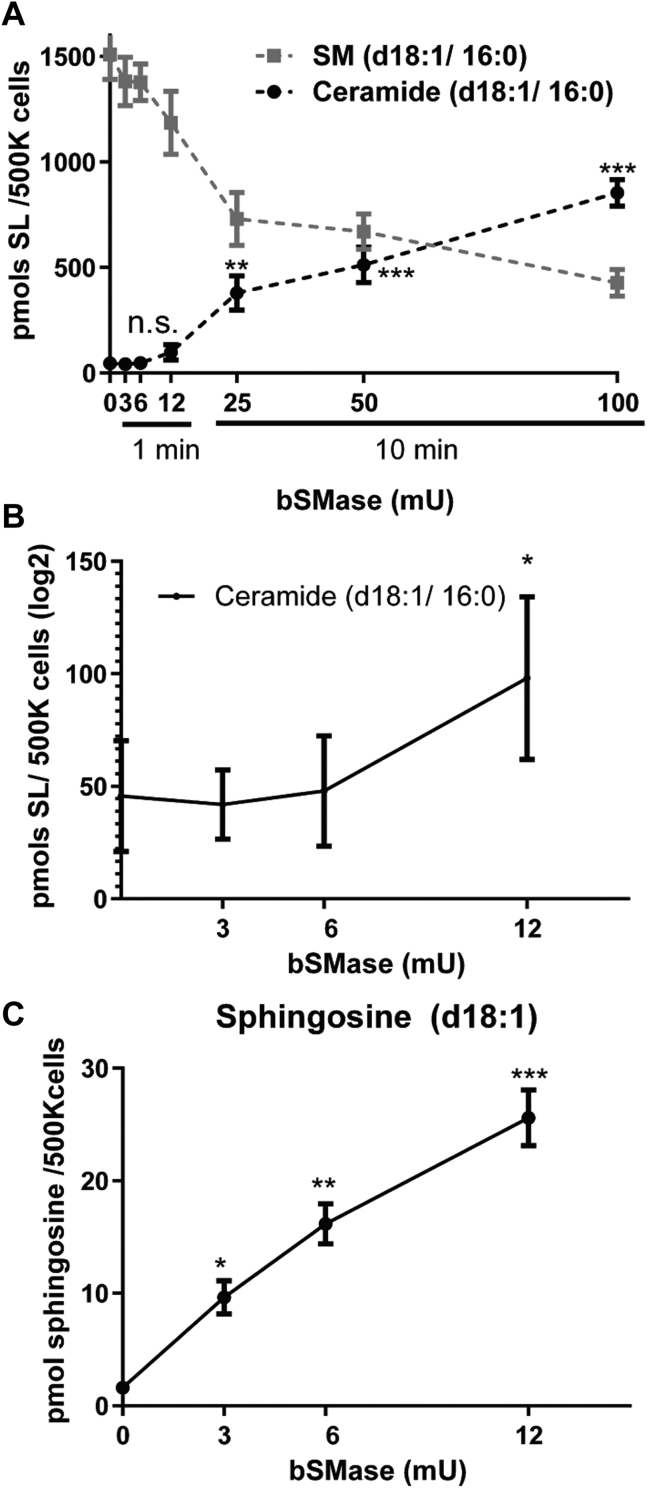
Limits of detection of PM-Cer. A: HeLa cells were treated with bSMase for increasing doses and times, washed and fixed, and sphingomyelin (SM) and ceramide were measured by LC-MS. B: HeLa cells were treated with low doses (0, 3, 6, and 12 mU bSMase) for 1 min, bSMase was washed, cells were fixed, and ceramide was measured by LC-MS. C: PCDase was added to the fixed cells, and sphingosine was measured. PM-Cer was measured as the difference between cells not treated with pCDase (basal) and pCDase-treated cells. Statistics: One-way ANOVA post hoc Tukey test. n.s. = not significant; ∗*P*<0.05; ∗∗ *P*<0.01; ∗∗∗ *P*<0.001. bSMase, bacterial sphingomyelinase; pCDase, recombinant bacterial ceramidase; PM-Cer, plasma membrane ceramide.

The previous experiments were performed using conditions where the whole accessible sphingomyelin at the plasma membrane is expected to be hydrolyzed. However, in physiological conditions, such as those where cells respond to chemotherapy or receptor-mediated activation of sphingomyelinases, it is reported that around 5%–20% of the total sphingomyelin is hydrolyzed (33, 34). To determine how sensitive this assay is, we treated cells with increasing amounts of bSMase for 1 or 10 min and measured the amounts of sphingomyelin and ceramide (Fig. 6A). At very low amounts and short treatment times, changes in sphingomyelin and ceramide were barely or not detected (Fig. 6A, B). However, using pCDase, under these conditions, we were able to find a linear response between the amount of bSMase and sphingosine production (Fig. 6C). These results show that changes in PM-Cer content are masked by total (PM plus intracellular) ceramide content. By selectively hydrolyzing PM-Cer into sphingosine, the assay is able to overcome this noise in the system, allowing a quantitative measurement of the increase in ceramide in the plasma membrane at very modest hydrolysis of plasma membrane sphingomyelin.

### Dose-response with doxorubicin

**Fig. 7:**
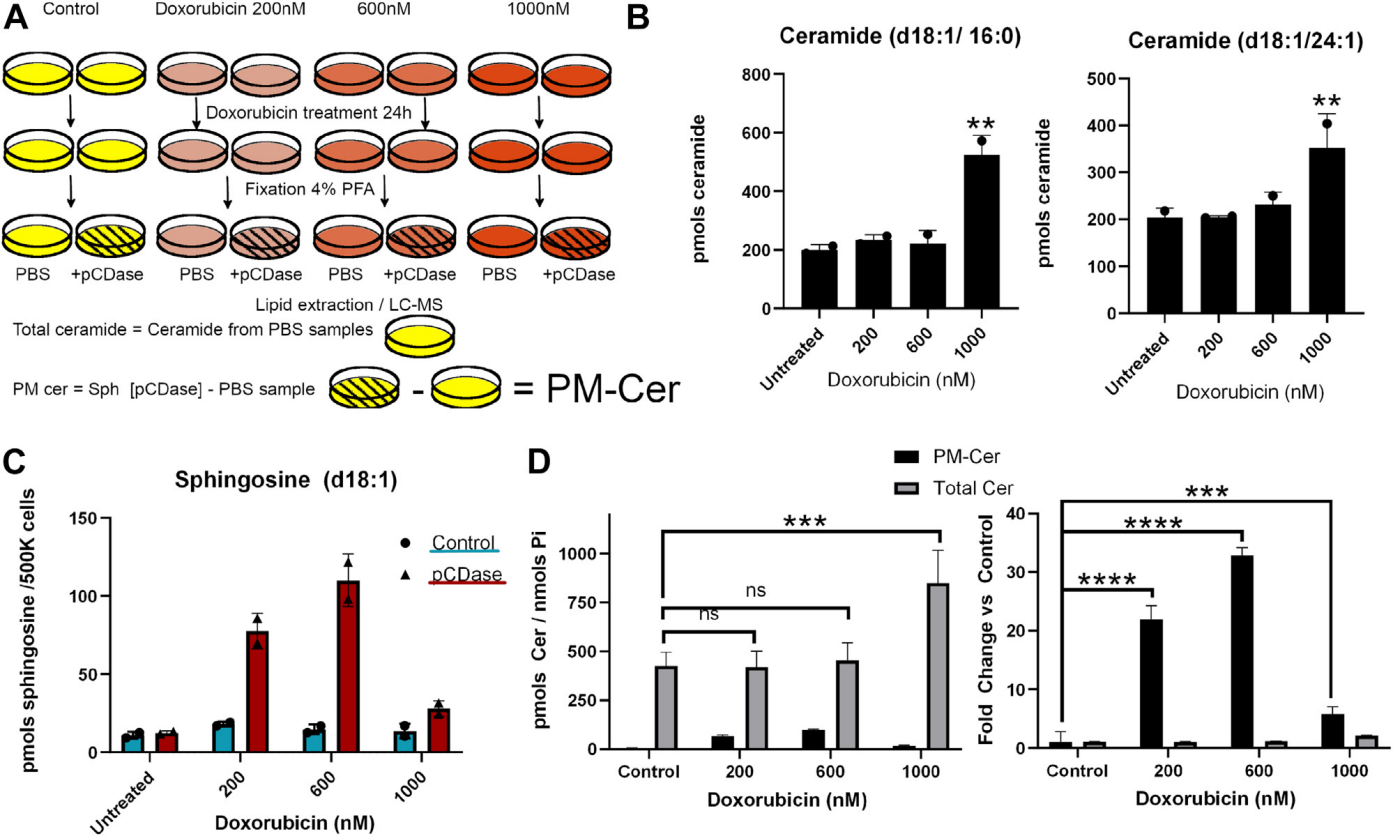
Detection of two pools of ceramide: PM-Cer at low doxorubicin doses and intracellular ceramide at higher doses. A: HeLa cells were treated with doxorubicin at a final concentration of 0, 200, 600, and 1000 nM for 24 h. Each condition was plated in duplicate, where one replicate was treated with vehicle (PBS) and the other one with pCDase. Cellular ceramides were measured from pCDase untreated dishes (results in B), and PM-Cer was calculated as the difference in sphingosine levels between the pCDase treated and the nontreated replicates (results in C). D: The left panel shows the total pmol amount quantified for each ceramide pool and each doxorubicin treatment. The right panel shows the fold change of each pool of ceramide when compared to untreated cells. Statistics: (B) One-way ANOVA post hoc Tukey test. ∗∗ *P*<0.01; (D) Two-way ANOVA post hoc Tukey test n.s. = not significant; ∗∗∗ *P*<0.001; ∗∗∗∗ *P*<0.0001. pCDase, recombinant bacterial ceramidase; PM-Cer, plasma membrane ceramide.

Using anticeramide antibodies, we and others have shown that doxorubicin treatment induces ceramide levels (23) and part of this ceramide has been located at the plasma membrane. In a previous publication, we showed that sublethal concentrations of doxorubicin (<800 nM) increased nSMase2 gene expression and ceramide levels, and this resulted in the loss of cell adhesion and an increase in cell migration (23). Ceramide at the plasma membrane was detected after 24 h of doxorubicin treatment. However, other works, using higher concentrations of doxorubicin (≥1000 nM) showed that doxorubicin increased ceramide and cell death. It was not clear if these two biologies of ceramide came from topologically identical or distinct pools. Here, we used the newly developed method to assess the levels of PM-Cer in response to sublethal and lethal levels of doxorubicin (Fig. 7A). As shown in Fig. 7B, sublethal doses of doxorubicin (200 and 600 nM) did not increase ceramide levels when these were measured in total cell lysate. However, as reported by us and others, lethal amounts of doxorubicin increased total ceramide levels (34). When only PM-Cer was measured, we detected ceramide production at the plasma membrane at sublethal doxorubicin but not at lethal doses (Fig. 7C). These results suggest that at least two different pools of ceramide are involved and that the PM-Cer is a minor component of the total cellular ceramide, yet changes after doxorubicin represent a significant fold change in the PM-Cer; for example, at the 600 nM doxorubicin, there was a greater than 30-fold increase in PM-Cer, although this amount of ceramide did not impact the total pool of cellular ceramide (Fig. 7D).

## DISCUSSION

**Fig. 8:**
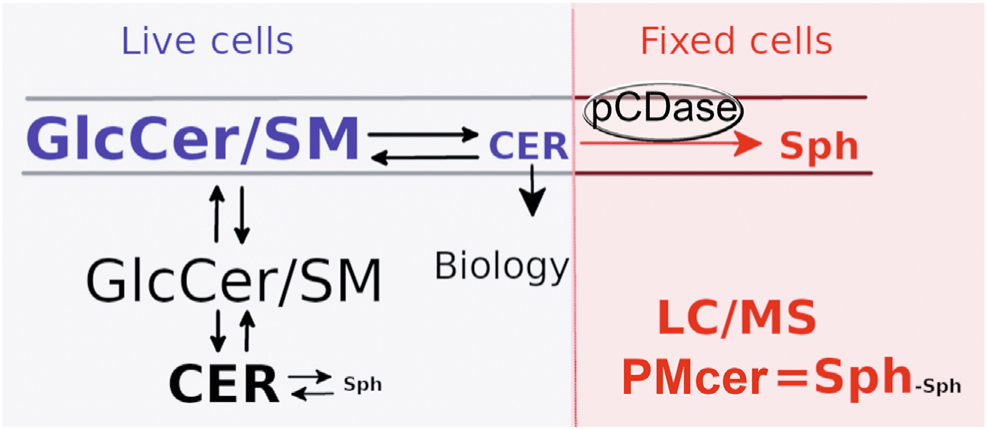
Measuring PM-Cer by converting it into sphingosine in fixed cells. Sphingolipid metabolism is present in many cellular membranes, where the large bulk of Cer is intracellular, being PM-Cer a small percentage of the total cer. The different compartments for ceramide (endoplasmatic reticulum, Golgi apparatus, plasma membrane, endosomal-lysosome, and mitochondria) are dynamic and interconnected by transport and metabolism (*blue*). Ceramide is significantly elevated in intracellular membranes, making its detection in the plasma membrane difficult. Cells are chemically fixed to stop metabolism, lipid transport, and membrane trafficking (*red*). pCDase is applied in the cell media and only acts on the plasma membrane to generate sphingosine from PM-Cer at a 1:1 M ratio. Intracellular ceramide is unaffected by pCDase treatment. Therefore, PM-Cer is calculated as the sphingosine generated from the pCDase-treated sample minus the sphingosine from the pCDase-untreated replicate. PM-Cer, plasma membrane ceramide; pCDase, recombinant bacterial ceramidase.

In this manuscript, we report on the first method to quantify ceramide in a specific compartment, the plasma membrane (PM-Cer). This method takes advantage of exogenously applied bacterial pCDase to access selectively PM-Cer, without affecting intracellular ceramide pools. Thus, pCDase hydrolyzed PM-Cer into sphingosine in a nearly quantitative manner. Upon 1 h of pCDase treatment, all accessible ceramide was hydrolyzed to sphingosine, and lipids were collected and extracted using conventional lipid extraction procedures. Sphingosine was then measured by LC-MS, and since endogenous sphingosine is much lower than PM-Cer, the biological variations in sphingosine levels did not interfere with the quantification (Fig. 8). The amount of endogenous sphingosine is calculated from an identical sample that is not treated with pCDase. To account for this endogenous sphingosine, this is then subtracted from the newly pCDase-generated sphingosine. Finally, PM-Cer is calculated as the generated sphingosine in a 1:1 M ratio. During treatment with pCDase and to avoid sphingolipid metabolism, membrane trafficking, and lipid transport between the plasma membrane and internal organelles, cells were chemically fixed with paraformaldehyde. This method allows quantifying PM-Cer with high sensitivity (∼0.5 pmol/500K cells or normalized by inorganic phosphate, ∼0.0.25 pmols/ nmol Pi; which represents around 0.1% of the total cellular ceramide). The assay allows high reproducibility and the screening of tens of different conditions simultaneously and in a short period (2 h).

In recent publications, we have highlighted the important role of PM-Cer in cell (2, 35). For example, the action of bSMase, added exogenously, resulted in dephosphorylation of ezrin-radixin-moesin proteins within 2–10 min, suggesting local action of the generated ceramide. Moreover, in response to the DNA damaging agent doxorubicin, HeLa cells generated ceramide, most likely in the plasma membrane as detected by validated anticeramide antibody, and this ceramide triggered the activation of a network of proteins in cell adhesion and cell migration (23). Other studies also have invoked PM-Cer in cellular signaling, and although PM-Cer was not directly measured, its role was supported by detecting activation of acid sphingomyelinase at the plasma membrane and by employing anticeramide antibodies and demonstrating binding to the plasma membrane (36, 37).

One of the limitations to measuring changes in signaling ceramide in the plasma membrane arises from the apparent very low levels of ceramide in the plasma membrane when compared to the total cellular ceramide. From the results in this work, HeLa cells contain only ∼0.1% of the total ceramide in the plasma membrane. However, upon physiological stimulation, this can rise to 5%–10% of total ceramide. Still, these amounts might fall within the standard deviation of total cellular ceramide, making this increase in ceramide statistically hard to validate. In addition, the dynamics of other pools of ceramide may mask the increase in the PM-Cer when total cellular ceramide is measured; e.g., if ceramide simultaneously decreases by even a small fraction in other pools. Here, we report the first method for quantifying the amount of ceramide in the plasma membrane in cultured cells. The method presented in this work bypasses these caveats by only targeting PM-Cer, and it is insensitive to the oscillations of the intracellular bulk. Endogenous cellular sphingosine is very low compared to ceramide (less than 5%), and a small increase of PM-Cer can therefore translate into a several-fold increase in sphingosine; thus, the variation of basal sphingosine between replicate samples is negligible compared to the sphingosine originating from hydrolysis of PM-Cer. The results in Fig. 7D illustrate the power of the study in detecting stimulus-induced formation of PM-Cer whereby Doxorubicin did not show much effect on total cellular ceramide, yet it induced more than 20- and 30-fold increases in PM-Cer at 200 and 600 nM, respectively.

Another key point that allows the success of this method is applying the ceramidase onto chemically fixed cells. This allows the conversion of PM-Cer into sphingosine without further metabolism of the generated sphingosine and without any enzymatic activity interfering with this reaction. As mentioned before, chemical fixation is commonly used in histochemistry to preserve the cellular structure. The effects on the cellular lipid structure have not been addressed. Here, we demonstrated that, at least for 1 h after chemical crosslinking with paraformaldehyde, the lipid composition in the plasma membrane does not change, and pCDase can convert plasma membrane, but not intracellular ceramide, into sphingosine.

Our group introduced the use of bacterial ceramidase as a tool to hydrolyze ceramide in cultured cells (21). We demonstrated that *Pseudomonas aeroginosa* ceramidase (pCDase) hydrolyzed all the common ceramide species found in HeLa cells, from C14 to C26 ceramides, with or without unsaturated acyl fatty acid chains. PCDase can be easily purified in *E.coli* BL21 in large amounts by His-tag affinity columns and stored in 50% of glycerol at −80C for years without losing activity. The pCDase is added in excess to ensure maximum activity during the 1 h of the assay. To confirm this, we added extra fresh pCDase and observed no additional ceramide was hydrolyzed (data not shown). Thus, the economical source of pCDase and the simplicity of the protocol also allows mass screening of samples.

It is important to mention that pCDase can target plasma membrane ceramide independently if this has been generated at the outer or the inner leaflet. In fixed cells, where any putative lipid transport protein is inactivated, ceramide will still equilibrate rapidly between the two layers. Thus, pCDase will hydrolyze ceramide at the outer leaflet, while the inner leaflet ceramide will flip flop in constant equilibrium to the outer side and be hydrolyzed until all plasma membrane ceramide is consumed.

A critical point in this approach is to ensure the quantitative conversion of PM-Cer to Sph. In fixed cells, PM-Cer is converted to sphingosine. For example, as shown in Fig. 4, bSMase generates ∼1000pmol of ceramide. In fixed cells, pCDase hydrolyzes ∼600pmols of ceramide [measured from Ceramides (d18:1/16:0) and (d18:1/24:1), the two main species of sphingomyelin in HeLa cells. Other species were not measured in this experiment, but the contribution to mass is negligible (21)], which is transformed to sphingosine, without undergoing further metabolism. This contrasts with the unfixed cells, where hydrolysis of PM-Cer is not translated to an equal molar amount of sphingosine but to an increase in S1P. Moreover, total ceramide levels do not drop, probably due to the recycling of part of the generated sphingosine back to ceramide and the upregulation of sphingolipid metabolism (e.g., de novo synthesis) resulting from the dramatic changes in sphingolipid content upon the pCDase action. To explain this discrepancy, if we assume that pCDase generated a similar amount of sphingosine in viable cells, we can account only for 150 pmols of sphingolipid in the form of S1P. The unaccounted amount, approximately the resting 450 pmols of sphingolipid, followed different metabolic paths: one part might be secreted into the media (as reported in S1P (25)) and the rest possibly underwent further metabolism. For example, S1P can be degraded by the S1P lyase (29). Sphingosine can be reacylated by ceramide synthases to ceramide (1). The cell could also respond to the newly generated sphingosine and S1P by regulating the de novo pathway, which could explain the small drop in total-measured ceramide levels. Importantly, all these become nonissues for PM-Cer quantification when cells are fixed.

Using this method in suboptimal conditions, the conversion of PM-Cer to sphingosine could become dependent on variations of external factors (e.g. time of the assay, temperature, amount of enzyme added, unsuspected factors that may regulate the kinetics of the enzyme, pCDase), and therefore, the measurement of sphingosine may not reflect PM-Cer content. This may not be obvious. Around three decades ago, our group developed an enzymatic assay to measure total cellular ceramide using DAG kinase (DGK) from *E. coli*. This enzyme can be used to convert ceramide and ^32^P-ATP, into ceramide-1-phosphate, then quantified by TLC and radioactive counts. Unfortunately, this was misused by others where DGK was used at low amounts that did not convert all ceramide. Because of this, factors that influenced DGK activity would result in a change in the conversion of ceramide in the assay itself. Used as such, the assay could not distinguish the effects on the DGK itself versus the effects on the actual levels of the substrates. This issue was discussed in detail in ref (38).

It is important to compare this method to other previously reported approaches. These are *1*) cell fractionation followed by gradient centrifugation and measuring ceramide content by mass spectrometry and *2*) ceramide antibodies. In the first approach, membrane purification protocols are subjected to long procedures (from a few hours to 24 h for sample preparation, but the whole process might require several days). Fractionation requires cell disruption, mixing membranes in microsome preparations and releasing metabolic enzymes to the cell lysate, which might modify membrane sphingolipid composition. Moreover, these protocols require a large number of cells (for HeLa cells, we needed to use 20–30 millions / condition (2, 23)) and tedious sample manipulation (sucrose or optiprep gradient preparation, manual fraction collection, organelle identification by Western blot, and each fraction needs to be extracted for lipid quantification by mass spectrometry), limiting the analysis to a few samples (2–4), and thus not suitable for screening, time-course, or dose-effect–dependent analysis. In our experience, this approach contains many points for the potential introduction of noise and variability (such as released endogenous enzymes and organelle cross-contamination). The quantification of ceramide from the different fractions is also problematic. Fractions are highly diluted in a high-density matrix, giving a poor signal to a noisy mass spectrometry ratio. Results from gradient fractionation protocols should be used to compare qualitative lipid changes between samples, but data should not be used for absolute quantification.

Ceramide antibodies allow the detection of ceramide in multiple samples and with a minimal number of cells, for example, we regularly use 35 mm dishes with 10.000 cells/ dish. Ceramide antibodies give the advantage of visualizing ceramide detection in individual cells. The signal is qualitative and needs to be compared to control samples. The signal intensity will vary from experiment to experiment, and it cannot be directly compared between experiments. One of the main concerns with ceramide antibodies is the lack of consensus in protocols and interpretation of the results. For example, ceramide antibodies have been described to stain only the outer leaflet of the plasma membrane (2) whereas other studies show intracellular membranes (20). By far the most important concern is specificity; a study comparing several of currently used ceramide antibodies showed that each ceramide antibody recognized also different lipids other than ceramide (39). Of the several ceramide antibodies used in the literature, only two of them have published validation experiments on cells (2, 20). Our group reported that from several ceramide antibodies tested, only two responded to sphingomyelin hydrolysis in the plasma membrane, whereas others did not respond to this obligate increase in ceramide (hydrolysis of even 10% of plasma membrane sphingomyelin generates several fold increase in total cellular ceramide (2, 35).

The lack of a quantitative assay has prevented the thorough study of PM-Cer, its generation, and its metabolism. Relying on measuring total cellular ceramide is not adequate as it is now apparent (e.g., see Figs. 6 and 7) that PM-Cer under resting conditions is very low (less than 1% of total ceramide), and the increases can be masked when we measure total cellular ceramide. This quantitative assay now allows us to investigate key questions on ceramide formation and function that have been unmanageable thus far.

We propose that the use of this method will force a re-examination of the regulation and role of PM-Cer in signaling. Often ceramide signal is measured in the total cell lysate. As mentioned before, this signal is altered by the fluctuations of other ceramide pools. Assignment of one or another pool is often based on genetic and pharmacological manipulation of the sphingolipid enzymes. However, the distinct pathways of ceramide metabolism are not independent but rather interconnected. Using doxorubicin as an example, we already detected the existence of two distinct elevations of ceramide, one in the plasma membrane, and a later one from intracellular ceramide. This method opens new avenues to explore ceramide signaling in specific compartments. Our group is already interested in and developing tools to analyze ceramide in other compartments, trying to understand and consolidate the different reported ceramide-related biologies with the existing ceramide pools and biosynthetic ceramide pathways.

## Data availability

Mass spectrometry raw data will be shared upon request: Daniel Canals, Stony Brook University, Cancer Center. MART building. Daniel.Canals@stonybrookmedicine.edu

## Conflict of interest

The authors declare that they have no conflicts of interest with the contents of this article.
